# Neovascular Glaucoma: A Retrospective Review from a Tertiary Eye Care Center in Mexico

**DOI:** 10.5005/jp-journals-10028-1222

**Published:** 2017-08-05

**Authors:** Gabriel Lazcano-Gomez, Jeffrey R Soohoo, Anne Lynch, Levi N Bonell, Karina Martinez, Mauricio Turati, Roberto Gonzalez-Salinas, Jesus Jimenez-Roman, Malik Y Kahook

**Affiliations:** 1 Research Scholar, Department of Ophthalmology, School of Medicine, University of Colorado, Colorado, USA; 2 Assistant Professor, Department of Ophthalmology, School of Medicine, University of Colorado, Colorado, USA; 3 Associate Professor,Department of Ophthalmology, School of Medicine, University of Colorado, Colorado, USA; 4 Professional Research Assistant, Department of Ophthalmology, School of Medicine, University of Colorado, Colorado, USA; 5 Fellow, Department of Glaucoma, Asociacion para Evitar la Ceguera en Mexico APEC, Mexico city, Mexico; 6 Professor, Department of Glaucoma, Asociacion para Evitar la Ceguera en Mexico APEC, Mexico city, Mexico; 7 Professional Research Assistant, Department of Glaucoma, Asociacion para Evitar la Ceguera en Mexico APEC, Mexico city, Mexico; 8 Chief, Department of Glaucoma, Asociacion para Evitar la Ceguera en Mexico APEC, Mexico city, Mexico; 9 Professor, Department of Ophthalmology, School of Medicine, University of Colorado, Colorado, USA

**Keywords:** Education, Mexico, Neovascular glaucoma, Outcomes, Socioeconomic.

## Abstract

**Aim:**

To describe the demographic characteristics, ocular comorbidities, and clinical outcomes of patients with neovascular glaucoma (NVG) and to determine the number of patients who returned for a follow-up eye examination.

**Materials and methods:**

We examined the clinical data of patients with NVG, who attended a glaucoma clinic between July 2010 and November 2014. We collected information on the demographic characteristics of the patients to include the level of education, ocular comorbidities, NVG stage, visual acuity, glaucoma medications, intraocular pressure (IOP), and the number of patients who had a follow-up ocular examination at month 1, 3, 6, and 12.

**Results:**

Data from 350 patients (473 eyes) with NVG were collected. We found 91% of the cohort had proliferative diabetic retinopathy (PDR). We found blindness in both or one eye in 14% and 31% of the cohort respectively. Low vision was found in both or one eye in 14% and 32% of the eyes respectively. By 6 months follow-up, only 32% of the patients were seen at our clinic and by 12 months follow-up, this number decreased to 15%. Around 60% of the patients were on no IOP lowering drugs at the first visit. We found 53% of the cohort had an incomplete elementary school education.

**Conclusion:**

The results suggest that advanced NVG is a significant ocular problem for patients referred to our clinic with just over half of the patients presenting as blind. We also found that several socioeconomic factors that had an important role in the development of PDR and NVG, specifically, educational status.

**Clinical significance:**

We described the characteristics of a large cohort of patients with very advanced NVG, reflecting the fact that the strict control of the underlying disease must be the main goal of the Mexican national health system.

**How to cite this article:**

Lazcano-Gomez G, Soohoo JR, Lynch A, Bonell LN, Martinez K, Turati M, González-Salinas R, Jimenez-Roman J, Kahook MY. Neovascular Glaucoma: A Retrospective Review from a Tertiary Eye Care Center in Mexico. J Curr Glaucoma Pract 2017;11(2):48-51.

## INTRODUCTION

Neovascular glaucoma (NVG) is defined as severe glaucoma associated with the presence of new iris vascularization (NVI) or angle (NVA) vessels.^1^ Patients with NVG typically present with a very high intraocular pressure (IOP) and hyphema.^2^ Once NVG develops and IOP becomes elevated, treatment becomes challenging and the outcome is often irreversible visual loss.^3-7^ Investigators from the United States of America and China have studied patients with advanced NVG and described central retinal vein obstruction (CRVO), proliferative diabetic retinopathy (PDR) and ocular ischemic syndrome as major comorbidities that place a patient at risk for adverse NVG-related ocular complications.^8-9^ However, apart from these studies, few investigators have reported the clinical characteristics of patients with NVG and their outcomes. Moreover, there has been no comprehensive review of patients with NVG from Latin America where a large proportion of the patients with diabetes have diabetic retinopathy (DR).^10^

The purpose of this study is to: (1) Describe the demographic characteristics, ocular comorbidities and clinical outcomes of patients with NVG and (2) determine the number of patients who returned for a follow-up eye examination. To address these aims, we conducted a retrospective cohort study of patients with advanced NVG who received care at a tertiary eye care center in Mexico City.

## MATERIALS AND METHODS

This retrospective chart review was approved by the ethics committee of the Asociacion para Evitar la Ceguera en Mexico (APEC), a referral center for complex eye diseases located in Mexico City. We examined the clinical data of all patients who attended the glaucoma clinic at APEC between July 2010 and November 2014. We collected information on the demographic characteristics of the patients to include: Age, gender, and level of education (categorized as incomplete elementary school education, elementary school, high school, and college/degree). We documented if the patient had CRVO, PDR, ocular ischemic syndrome, uveitis or other ocular comorbidities. The NVG stage was recorded and defined as:

*Stage* I: NVI/NVA with normal IOP,

*Stage II:* NVI/NVA, open angle and IOP above normal limits, and

*Stage III:* Angle-closed glaucoma with high IOP (described in detail elsewhere).^11^

Visual acuity was categorized as normal vision (20/60--20/15; Snellen), low vision (20/400 - 20/80), and blind (<20/400).^12^ The number of glaucoma medications and IOP measurements at baseline were also recorded. In addition, we recorded the number of patients from the cohort who had a follow-up ocular examination at month 1, 3, 6, and 12.

## Statistical Analysis:

The statistical analysis was performed using SAS software version 9.4 (SAS Institute Inc., Cary, NC). We generated descriptive statistics using univariate analysis.

## RESULTS

Data from 350 patients and 473 eyes with NVG were collected. The overall characteristics of the cohort (n = 350) at the first visit and of the eyes (n = 473) are shown in Tables 1A and 1B respectively. As shown in Table 1A, we found that a higher percent of the patients were male and 53% of the cohort had an incomplete elementary school education. The mean age was 61 years. The most frequent ocular comorbidity associated with NVG was PDR followed by CRVO and uveitis. We found no patients with ocular ischemic syndrome.^13^ By 6 months follow-up visit, only 32% of the patients were seen at our clinic and by 12 months follow up, this number decreased to 15%. We show in Table 1B the clinical characteristics of the 473 eyes included in this study. It is noteworthy that 49% of 473 eyes had NVG stage 3 and 55% were blind at baseline. We found that 60% of 473 eyes were under no treatment for glaucoma at the time of the baseline visit.

Table 2 demonstrates the clinical characteristics of the patients with NVG in both eyes (n = 123). As demonstrated, we found evidence of blindness in both eyes or one eye in 14% and 31% of the cohort respectively. Low vision was found in both or one eye in 14% and 32% of the eyes respectively. Only 7% of the eyes with bilateral NVG had normal vision.

**Table 1A:** **Table 1A.** Characteristics of cohort at baseline (n = 350 patients)

*Mean age (years) ± SD*		*60.9 ± 12.6 years*					
				*n*		*%*	
Gender		Male		220		62.86	
		Female		131		37.43	
Education		Incomplete elementary school		185		52.86	
		Elementary school		95		27.14	
		High school		45		12.86	
		College/Degree		25		7.14	
Ocular diseases (cause		PDR		318		90.8	
of NVG)		CRVO		25		7.14	
		Uveitis		5		1.42	
		Ocular surgery		2		0.57	
No. patients (follow-up)		Month 1		294		84.00	
		Month 3		210		60.00	
		Month 6		111		31.71	
		Month 12		53		15.14	

**Table 1B:** **Table 1B.** Clinical characteristics of the eyes (n = 473)

Neovascular glaucoma		1		58		12.26	
stage		2		183		38.69	
		3		232		49.05	
Mean IOP ± SD		36.93 ± 16.28 mm Hg					
Glaucoma medications		0		284		60.04	
		1		8		1.69	
		2		7		1.48	
		>3		174		36.79	
Visual acuity		Normal		76		16.07	
		Low		135		28.54	
		Blindness		262		55.39	

**Table 2:** **Table 2.** Clinical characteristics of the patients with neovascular glaucoma in both eyes (n = 123)

Blind in both eyes		17		14%	
Blind in one eye		39		31%	
Low vision in both eyes		17		14%	
Low vision in one eye		39		32%	
Bilateral normal vision		9		7%	

## DISCUSSION

We studied a large contemporary cohort of patients with NVG attending a tertiary eye care center in Mexico City. The most noteworthy finding of the study is that half of the eyes had stage 3 NVG and 55% of the eyes were blind at the baseline visit. Only 40% of the eyes were treated for their raised IOP at the baseline visit. These findings may reflect lack of education in this cohort as only 20% of the subjects included had completed more than elementary school education.

Proliferative diabetic retinopathy was the most common ocular disease found in this cohort with an incidence of 90.8%. This incidence is very high compared with the incidence of PDR described in other populations.^14-15^ Several studies from North America have described the prevalence of DR varying from 24.8% in Caucasian patients with type II diabetes mellitus (DM) to 41.0% in patients with type I DM, with a prevalence of nonprolif-erative retinopathy of 12.8% and proliferative retinopa-thy of 4.0%.^16,17^ Investigators from the United Kingdom studied a large cohort (n = 307,538) of diabetic patients and found 12.6-20.6% of eyes with no DR; 59.6-67.3% had nonproliferative DR; and 18.3-20.9% with active or regressed proliferative DR.^18^ In a similar population to our study, investigators with the Latinos Eye Study described 46.9% of 1263 patients with DM had DR, and that severe nonproliferative DR and proliferative DR were present in 4.4 and 6.1% of diabetics respectively. In addition, these investigators found a progression from nonproliferative DR to PDR and from nonproliferative PDR to PDR in 5.3 and 1.9% of participants respecitively.^19^ We propose that the high number of patients with PDR seen in our study may be the result of a referral bias as our eye clinic is a regional referral center for patients with complex eye diseases. Moreover, these results could also reflect the high incidence of type II DM in Latin America.^20^

Latin America is one of the regions with a highest increase in the incidence of type II DM and indeed type II DM has been described as a leading cause of death in Mexico.^21^ Previous studies in Mexico have reported a prevalence of DR ranging from 38.9% to 51%,^22,23^ with worsening of retinopathy in 20.6% of the patients with DR.^24^ Reports from Instituto Nacional de Estadistica y Geografia have suggested that the main factor that contributes to a high prevalence of DM and its ocular complications in Mexico is the low level of education with an average of 9 years of education completed per person.^25^ This was also a finding in our study where 80% of the patients had less than elementary school education. These findings also correlate with data from Proyecto Ver whereby income less than $20,000/year USD, a surrogate measure of educational status was associated with PDR.^26^

Mexico has been considered a country with a low proportion of resources allocated to health (6.2%) and health expenditure when compared with other countries of the Organization for Cooperation and Economic Development (OCDE).^27,28^ Diabetes mellitus represents the main economic burden within the health sector institutions with an annual expenditure for treating DR is reported to be 30,000 USD per patient.^29^ Many recommendations to improve this severe national health problem were proposed by "Foro de Alto Nivel Sobre Estrategias de Prevencion y Tratamiento de la Diabetes en Mexico" The discussions held at the event and the opinion of experts in the field have identified several recommendations that the Mexican state must provide to counteract this condition (described in detail elsewhere).^30^

As NVG is a highly challenging and unpredictable disease, a high index of suspicion of its development is mandatory. Early treatment of the underlying disease can reduce the development of NVG complications, such as high IOP, which is almost invariably the main factor in irreversible and massive visual loss.^31^

Notwithstanding the important findings of this study, there were some limitations. The main limitations were that we conducted a retrospective chart review and had a high rate of dropout , thereby, limiting the collection of follow-up data on this high risk cohort.

## CONCLUSION

The results of our study suggest that advanced NVG is a significant ocular problem for patients referred to our clinic with just over half of the patients presenting as blind. We suggest that PDR has a significant role in the development of NVG. We also found several socioeconomic factors that had an important role in PDR and NVG, specifically educational status. Indeed, a lower income or level of education can lead to underutilization of appropriate preventive care, and less use of health or eye care services. The ability to properly diagnose and treat NVG can be limited in resource poor developing countries, such as Mexico. Although medical treatments can help lower IOP, the main goal of NVG treatment is early diagnosis and treatment of the underlying disease prior to the development of elevated IOP.

## CLINICAL SIGNIFICANCE

We described the characteristics of a large cohort of patients with very advanced neovascular glaucoma, reflecting the fact that the strict control of the underlying disease must be the main goal of the national health system.
